# Optimal bladder diary duration for patients with suprapontine neurogenic lower urinary tract dysfunction

**DOI:** 10.1590/S1677-5538.IBJU.2015.0292

**Published:** 2016

**Authors:** Charalampos Konstantinidis, Zisis Kratiras, Michael Samarinas, Konstantinos Skriapas

**Affiliations:** 1 Urology & Neuro-urology Unit, National Rehabilitation Center, Athens, Greece; 2 Department of Urology, General Hospital of Larissa “Koutlibanio”, Larissa, Greece; 3 Department of Urology, University of Thessaly, Larissa, Greece

**Keywords:** Urinary Bladder, Pathology, Neurogenic Bowel, Lower Urinary Tract Symptoms

## Abstract

**Purpose:**

To identify the minimum bladder diary's length required to furnish reliable documentation of LUTS in a specific cohort of patients suffering from neurogenic urinary dysfunction secondary to suprapontine pathology.

**Materials and Methods:**

From January 2008 to January 2014, patients suffering from suprapontine pathology and LUTS were requested to prospectively complete a bladder diary form for 7 consecutive days. Micturitions per day, excreta per micturition, urgency and incontinence episodes and voided volume per day were evaluated from the completed diaries.

We compared the averaged records of consecutive days (2-6 days) to the total 7 days records for each patient's diary, seeking the minimum diary's length that could provide records comparable to the 7 days average, the reference point in terms of reliability.

**Results:**

From 285 subjects, 94 male and 69 female patients enrolled in the study. The records of day 1 were significantly different from the average of the 7 days records in every parameter, showing relatively small correlation and providing insufficient documentation. Correlations gradually increased along the increase in diary's duration. According to our results a 3-day duration bladder diary is efficient and can provide results comparable to a 7 day length for four of our evaluated parameters. Regarding incontinence episodes, 3 days seems inadequate to furnish comparable results, showing a borderline difference.

**Conclusions:**

A 3-day diary can be used, as its reliability is efficient regarding number of micturition per day, excreta per micturition, episodes of urgency and voided volume per day.

## INTRODUCTION

Lower Urinary Tract Symptoms (LUTS) secondary to neurological disorders appear to be a major health problem affecting patients worldwide (1). Voiding dysfunction has a remarkably negative impact on the quality of life and may lead to significant and life threatening complications (2), increasing simultaneously the economic burden for patients and health providers. The usual history-taking in patients with neurological disorders seems inadequate for the assessment of LUTS due to subjective factors. Several clinical trials targeting a more objective and accurate evaluation of LUTS have employed urinary diaries in order to identify voiding patterns and to quantify response to treatment. According to the International Continence Society, three types of urinary diaries have been defined (3): Micturition Chart, Frequency Volume Chart and Bladder Diary (BD).

Nevertheless, a validated standardized bladder diary in terms of diary format, duration and evaluated parameters, has not yet been established (4). As a result, a BD with an optimum duration, which may ensure reliable results having simultaneously an acceptable level of patient's compliance, remains a dispute.

Recent and former literature has studied mostly non-neurogenic populations, having only rare references focusing on neurogenic lower urinary tract dysfunction in patients suffering from suprapontine pathology. So, the aim of our study was to determine the optimal length of a BD in terms of test reliability in this special cohort of patients. Analyzing the data derived from a 7-day bladder diary we evaluated five parameters seeking the optimal length for each one, assuming that the 7-day duration urinary diary is the reference point in the assessment of LUTS (5, 6).

## MATERIALS AND METHODS

### Type of the study

Prospective, quantitative survey from January 2008 to January 2014, involving 285 consecutive patients.

### Inclusion/exclusion criteria

Inclusion criteria were treatment-naive patients of both genders, older than 20 years, suffering from suprapontine pathology caused either by traumatic brain injury or by cerebrovascular accident (CVA). Patients should be mentally fit with the ability to understand and communicate and the appearance of their LUTS should prelude at least 2 weeks before the integration on this study. For the mental evaluation of our subjects, Mini Mental State Examination was used (7). Exclusion criteria were multiple sclerosis, confused state, depression, dementia, bladder cancer, pregnancy, bladder stones, bladder outlet obstruction, diabetes, and urinary tract infection. Furthermore, subjects with a previous history of stress incontinence were also excluded from this study, in order to avoid the real cause of present LUTS to be confused and mixed. Additionally, male individuals over 50 years of age were excluded, considering that the co-existing benign prostate hyperplasia might interfere and distort our results. Moreover, after a proper vaginal examination, female patients suffering from any kind of vaginal pro-lapse were also excluded from the study, since vaginal prolapse might cause bladder outlet obstruction distorting our results.

### Process

All patients at the initial visit were clinically evaluated with physical examination, history report, urine analysis and urine culture, abdominal ultrasound and post-void residual volume evaluation. Patients received a bladder diary form and were requested to complete it for 7 consecutive days beginning with the first morning void. Patients were advised to record the precise time and volume of each void. The beginning of every day was defined at 08.00a.m. and the end at 07.59a.m. next morning (8).

Additionally, the episodes of urgency and incontinence have also been recorded. The evaluated parameters of our study were: micturitions per day (voiding frequency), excreta per micturition (mL per void), episodes of urgency and incontinence and voided volume per day.

#### Statistical analysis

At the second visit, we analyzed the diaries and averaged the records over 2 to 7 days. The records of each subject were considered as dependent scale parameters and the total average of the 7 days records as the reference point of our comparisons in terms of test reliability.

As a result we used paired t-tests, in order to compare the averaged records of different days (2-6 days) to the total 7 days records for each patient's diary. We sought to identify which day's mean had no statistically significant difference with the 7 days average in terms of each assessed parameter. In all comparisons p value<0.05 was considered as statistically significant. SPSS version 20.0 was used for the statistical analysis.

## RESULTS

From the total of 285 patients, 122 (42%) patients were excluded while 163 subjects (58%) met the inclusion criteria and were prospectively enrolled in the study. Among the 122 patients, 12 (9%), 31 (25%) and 30 (24%) were excluded from the study due to urinary infection, diabetes and mental instability respectively. In addition, 40 (32%) male patients over 50 years old were also excluded. The 94 male and 69 female patients enrolled in our study ranged in age from 23 to 68 years, with a mean age of 43.6 years.

The data retrieved from the diaries were summarized, averaged and the descriptive statistics along with the general characteristics of our cohort are provided in Tables 1a and 1b. Correlations gradually increased along the increase in the diary's duration reaching the maximum on the day 6. Table-2 summarizes the correlations of the different diary's duration records with the 7 days average.

**Table 1a:** Descriptive statistics of the cohort. Average data derived from the completed diaries.

	Number of patients	Mean	Std. Error	Std. Deviation
Av. Micturitions	163	9.92	0.19	2.44
Av. Excreta/micturition (mL/void)	163	121.83	1.45	18.44
Av. Urgency Incidents	163	5.10	0.06	0.73
Av. Incontinence episodes	163	4.99	0.07	0.92
Av. Total voided volume (mL)	163	1140.41	13.14	167.30

**Table 1b:** Patient's general characteristics

	Male	Female	Total
Patients (n)	94	69	163
Mean age (yrs)	30.3	61.7	43.6
Brain trauma (n)	59	26	85
Cerebrovascular accident (n)	35	43	78

**Table 2:** Correlations of different diary's length with the 7-day duration diary for the evaluated parameters (r values).

	Day 1	Day 2	Day 3	Day 4	Day 5	Day 6
Micturitions	0,832	0,800	0,929	0,974	0,972	0,947
Excreta/micturition	0,745	0,866	0,891	0,839	0,970	0,990
Urgency episodes	0,609	0,669	0,822	0,922	0,961	0,979
Incontinence episodes	0,664	0,707	0,798	0,906	0,968	0,969
Total Urine	0,823	0,955	0,956	0,902	0,973	0,980

According to our test results, a 1-day and 2-day length bladder diary could not provide results comparable to a 7-day diary in any parameter, since the differences that arose using the paired t-test between these durations were statistically significant. Thus, regarding micturitions per day, excreta per micturition, urgency episodes and total voided volume the 3-day records were not significantly different than the 7 days records (p>0.05). This absence of statistical significance was interpreted as a failure to establish a true difference between the 3-day length and the 7-day length records for 4 of the 5 evaluated parameters. As a result, the 3-day diary could provide data comparable to the 7-days in terms of these parameters. As for the incontinence episodes the 3-days length was borderline significantly different from the 7-day length (p=0.044), becoming statistically non significant from day 4 and on. Table-3 summarizes the results of the comparisons between the different diary's lengths. Comparisons between 1-day to 7-day and 6-day to 7-day duration were omitted from the Table in order to become less complicated. Mean differences along with 95% confidence intervals and P values of each comparison, between durations, are presented in the Table.

**Table 3:** Results of the comparisons between different diary's length (comparison day1 versus day 7 and day 6 versus day 7 were omitted from the Table). Mean difference, 95% confidence intervals and p value of each comparison are provided.

		Day 2 vs. Day 7	Day 3 vs. Day 7	Day 4 vs. Day 7	Day 5 vs. Day 7
	Mean diff.	-0.29	0.13	-0.012	0.049
Micturitions	95% Cl	(-0.522, −0.056)	(-0.014, 0.274)	(-0.1, 0.075)	(-0.041, 0.138)
	P value	0.016	0.077	0.776	0.282
	Mean diff.	2.50	-1.75	-0.27	0.07
Excreta per micturition (mL/void)	95% Cl	(0.252, 4.757)	(-3.551, 0.035)	(-1.956, 1.413)	(-0.619, 0.778)
	P value	0.03	0.055	0.75	0.822
	Mean diff.	0.12	0.06	-0.03	0.01
Urgency episodes	95% Cl	(0.013, 0.237)	(-0.005, 0.126)	(-0.084, 0.022)	(-0.021, 0.045)
	P value	0.028	0.071	0.254	0.489
	Mean diff.	0.16	-0.11	-0.06	0.027
Incontinence episodes	95% Cl	(0.019, 0.306)	(-0.207, −0.031)	(-0.126, 0.003)	(-0.009, 0.065)
	P value	0.027	0.044	0.061	0.141
	Mean diff.	-12.15	-9.32	-8.65	4.30
Voided volume (mL)	95% Cl	(-22.628, −1.633)	(-17.062, 0.473)	(-21.402, 4.092)	(-0.701, 10.304)
	P value	0.024	0.064	0.182	0.159

## DISCUSSION

BD's are widespread used by clinicians in the assessment of voiding dysfunction, providing real-time documentation of LUTS and voiding habits in the patient's environment during everyday life. BD's reduce the recall bias (9–11) that affects questionnaires like history-taking, providing simultaneously objective evidence for the evaluation of several therapeutic interventions. Urodynamic studies (UDS) have been considered as the mainstay in the diagnostic approach of patients suffering from LUTS and cannot be replaced by a diary. Nevertheless, voiding diaries are a useful tool in the initial management of LUTS providing information of daily bladder function, contributing to a more integrated interpretation of the UDS' results, particularly in terms of the functional bladder capacity (12).

No consensus has been reached yet for the optimal duration of BD since no sufficient data could be found in the literature. In several published reports, diaries' duration varies from 24 hours to 14 days. Wyman et al. (9) was the first who recommended that 7-day duration was sufficient to provide reliable documentation of urinary frequency and incontinence episodes in women. The most commonly recommended duration in literature is the 7-day diary (5, 6, 9, 13–15), covering both leisure and working activities. Nevertheless, several studies reported that a diary with less duration than 7 days could furnish comparably reliable results (16).

Nygaard and Holcomb (17) have suggested that a 3-day length is appropriate for trials evaluating stress incontinence, while Brown et al. (18) reported that, in patients suffering from urgency urinary incontinence, a 3-day duration diary could furnish reliable results in terms of daytime frequency, nocturia, urgency and incontinence episodes. In 2005, Dmochowski et al. (19) analyzed, reviewed and compared two large-scale randomized phase III clinical trials with patients suffering from overactive bladder, treated with transdermal oxybutynin. In both trials patient's symptoms were evaluated through voiding diaries with different durations. In trial A 520 patients documented their symptoms with a 7-day diary, while in trial B 361 patients documented their symptoms with a 3-day diary. Comparing the different durations, they concluded that 3-day diary appears to be equally effective to the 7-day, increasing potentially patient's compliance in clinical trials. Our results appear to be in accordance to the conclusion of the previously mentioned studies. Although the design and the cohort of our study was totally different, our results indicate that a 3-day diary length is adequate, effective and comparable to 7-day, providing credible results in patients suffering from suprapontine pathology in terms of the majority of the evaluated parameters.

Despite protracting the duration of the charts leads to an enhancement of the test reliability, prolonged studies might increase patient's burden leading to decreased compliance, a term described as “diary fatigue” (6, 20). Tincello et al. (21) announced that 90.7% of the patients completed a 3-day diary compared to only 50% who completed a 7-day diary, respectively. Shick et al. (16) reported that patient's compliance in BDs follows a logarithmic curve, in contrast to the reliability of the results which follows a bell-shaped curve. The intersection of these two curves represents the minimum duration of a diary which simultaneously provides acceptable reliability of the results.

In 2004, Ku et al. (22) prospectively enrolled 193 patients with incontinence and LUTS in a trial targeting to determine the appropriate duration of a diary assessing patient's compliance and burden. According to their results patient compliance was not compromised by the 7-day diary while patient's burden increases along the increase in the duration of the chart. Although patient's burden was not an evaluated parameter in the design of our study, we should mention the fact that the majority of our patients complained at the second visit about the duration of the BD, claiming that was rather inconvenient.

The majority of previous reports, focusing on the ideal duration of a BD, enrolled patients suffering from LUTS caused by several different conditions (diabetes, benign prostate hyperplasia, overactive bladder). In contrast to them, our study population was totally homogenous, since all subjects suffered from neurogenic urinary dysfunction secondary to suprapontine pathology. Thus, the data provided in the literature for this specific population are deficient and sparse. To the best of our knowledge, this is one of the first attempts enrolling such a pure cohort. In such cohort, the neurologic condition of patients is mainly responsible for their urinary dysfunction. As a result, patients' storage and voiding function are less influenced from social, behavioural or environmental factors. The clinical assessment of such patients could be very strenuous due to their neurological pathology and their consequent mobility problems. The majority of patients might be hospitalized for a long time; making their evaluation with the “classic” diary pattern, which includes leisure and working activities, unsuitable. According to our results a 3-day duration bladder diary is efficient and can provide results comparable to a 7-day length for 4 of our evaluated parameters. Only regarding incontinence episodes, 3 days seems inadequate to furnish comparable results, showing a borderline difference. In conclusion, 3-day bladder diary appears to be a quite useful and valuable tool in the evaluation of LUTS in this specific group of patients.

Nevertheless, our study has some limitations. Although it was prospective, we had not designed a second completion of the diaries by the same subjects, as a retest. This could have added concurrent validity to our results (23). Total fluid intake and patient's burden were not evaluated in the study. Moreover, we have to mention that our patients were not evaluated by UDS and that our study was based on the use of a self-recorded diary (20). Finally, we have to point out that the results of the current study cannot be applied to other voiding dysfunctions as only suprapontine neurogenic disorders were included.

## CONCLUSIONS

Three-day duration for bladder diary is as effective and sufficient as the 7-day, in at least 4 out of 5 evaluated parameters, in patients suffering from neurogenic urinary dysfunction due to suprapontine pathology. Thus, a 4-day length BD can provide comparable results in all parameters. In conclusion, the reliability of results was not compromised by the reduction in the number of days, clearly suggesting that the 7-day diary is unnecessarily protracted. Nevertheless, since LUTS derived from several different clinical conditions, it seems necessary that a validated standardized bladder diary in terms of diary format, duration and evaluated parameters needs to be developed in the future.
